# The trade of illicit cigarettes in Ghana: Insights from a policy synthesis and qualitative study

**DOI:** 10.18332/tpc/195578

**Published:** 2025-01-20

**Authors:** Arti Singh, Fiona Dobbie, Allen Gallagher, Hana Ross, Olivia A. Boateng, Divine Darlington Logo, Linda Bauld, Anna B. Gilmore, Ellis Owusu-Dabo

**Affiliations:** 1 School of Public Health, Kwame Nkrumah University of Science & Technology, Kumasi, Ghana; 2 University of Edinburgh, Edinburgh, United Kingdom; 3 Tobacco Control Research Group, University of Bath, Bath, United Kingdom; 4 University of Cape Town, Cape Town, South Africa; 5 Food and Drugs Authority, Accra, Ghana; 6 Ghana Health Service, Accra, Ghana

**Keywords:** cigarettes, Ghana, documents, illicit, stakeholder interviews

## Abstract

The illicit trade of tobacco products trade continues to challenge tobacco control efforts in several African countries, including Ghana. Ghana recently ratified the Framework Convention for Tobacco Control (FCTC) Protocol to Eliminate Illicit Trade in Tobacco Products (‘the Protocol’). This Protocol aims to eliminate all kinds of illicit tobacco. This study aims to enhance understanding of Ghana’s illicit cigarette market size, nature, and contributing factors. In doing so, it will help to inform future tobacco control policy. This study included a desk-based mapping of unpublished data looking at existing documents, reports, and websites (n=24 data sources) from 2012 to 2023 and semi-structured interviews with key informants representing government, civil society organizations, media, and international organizations (n=10). Stakeholders indicated the illicit cigarette market in Ghana to be a cross-border issue, with its extent ranging from 10% to 35% of the total cigarette market. Porous borders, lack of political interest, the tobacco tax structure, industry interference, and poor awareness and capacity of customs officials, were identified as contributing factors. Documents reviewed, highlighted the introduction of tax stamps in 2018, ratification of the Protocol in 2021, and the recent review of tobacco taxes as important steps taken by the government to address the illicit cigarette market. This study provides novel insights into the illicit cigarette market in Ghana and identifies major advances in the government’s strategy to address it. However, there remains a need for continuous monitoring of borders, alongside more investment in enforcement, national workshops, and training for customs and government officials on the current tax structure, industry tactics, and full Protocol implementation.

## INTRODUCTION

Illicit cigarette trade is a pressing issue with significant public health and fiscal policy implications. The Protocol to Eliminate Illicit Trade in Tobacco Products (‘the Protocol’) of the Framework Convention on Tobacco Control (FCTC) aims to eliminate all kinds of illicit tobacco^1^. This treaty provides mechanisms for stopping illicit tobacco trade by securing the supply chain (including through the adoption of tracking and tracing systems), mandating strong law enforcement, and boosting international collaboration^1^. Illicit cigarettes account for -up to 40% of the total tobacco market in some countries (e.g. Cameroon), and an estimated one out of every ten cigarettes and tobacco products consumed globally is illicit^2^.

Low- and middle-income countries (LMICs) continue to have a higher share of illicit cigarettes as compared to high-income countries (10.4% and 12.3%, respectively)^[Bibr cit0003]3^. In Africa, estimates for illicit tobacco account for over 43 billion sticks each year, with some countries accounting for as much as 41% (e.g. Cameroon) and 38% (e.g. Ethiopia), with the majority of countries at 25% (e.g. Algeria, Nigeria, South Africa, and Zambia)^4^. Illicit cigarettes are also particularly appealing to the region’s most price-sensitive and vulnerable populations, such as young people and individuals on low incomes^5^.

Ghana, one of the first countries to ratify the World Health Organization’s (WHO) FCTC in 2004, has a comparatively low smoking prevalence (<10%), with predominantly male smokers and significant mortality rates due to smoking ^6^. Although there is no active tobacco production in Ghana (British American Tobacco, BAT, ceased its local production in 2006), BAT continues to dominate sales of cigarettes and remains the dominant importer of cigarettes into the country via its manufacturing sites in Nigeria^7^. The distribution networks of Ghana’s leading tobacco companies are well organized in major urban cities, including Greater Accra, Takoradi, Kumasi, and Tamale. In Ghana, cigarettes are mostly sold at unlicensed and unregulated points of sale, such as traditional grocery retailers (commonly known as provision stores), street vendors, kiosks, and drinking bars^8^. A summary of tobacco use in Ghana is given in Supplementary file Material 1.

According to Euromonitor International, the illicit cigarette market in Ghana accounts for 39% of total cigarette sales in 2018 (and 35% in 2017)^8^. However, Euromonitor’s data has been criticized for its reliance on tobacco industry intelligence and the use of an opaque modeling process that may lead to biased estimates^9^. More recent estimates from an empty-pack survey on the illicit cigarette industry, conducted by the authors, reported a lower prevalence of 20% of the total market^10^. The tobacco industry consistently argues that increases in excise taxes (as well as the implementation of other tobacco control measures) will cause increases in illicit trade, often producing or commissioning greatly exaggerated estimates of illicit tobacco trade to support this narrative^11,12^.

Nevertheless, Ghana has made important strides in tobacco control: the implementation of an early advertising ban (1982), the passage of the Tobacco Control Act (in 2012), the prohibition of single-stick sales (2017), the implementation of graphic health warnings (2018), provision of tax stamps on tobacco products (2018)^13^, and, more recently, the ratification of the ITP (2021) and a review of tobacco taxes (at the time of data collection)^14^.

Single stick sales continue to be rampant in Ghana^15^, and cigarettes remain accessible and inexpensive (< US$1 per pack), with total excise taxes on tobacco products in Ghana accounting for only 31.8% of the average retail price^16^. Before March 2023, the *ad valorem* excise taxes (i.e. calculated as a proportion of product price) levied on cigarettes were at 175% of the customs, insurance, and freight (CIF) value (CIF is the value of an imported product as declared to customs upon entry into a territory).

However, since the CIF value is typically <10% of the retail price, the excise tax (the percentage of the retail price accounted for by excise) is small compared to the WHO standard for taxes (i.e. 70% of the overall excise taxes)^[Bibr cit0017]17^. In March 2023, the government of Ghana introduced the Excise Duty Amendment Bill 2023 (Act 1093) with revisions in the taxes on tobacco products^14^.

To date, the problem of the illicit cigarette trade in Ghana has received little attention in the literature, with only one scientific publication. This study intends to expand understanding of Ghana’s illicit tobacco market to the development and implementation of policy that is (better) matched to the needs of tobacco control (demand reduction) and the government’s income generation goal (illicit tobacco affects revenue generation). Also, to develop new tobacco control policies and further implementation of the Protocol, there is a need to understand the regulatory environment in Ghana. This study seeks to contribute to the evidence gap by enhancing understanding of:

- The extent and nature of the illicit cigarette market in Ghana;
- The tobacco industry’s involvement/influence in the illicit cigarette trade in Ghana; and
- The government’s response to illicit cigarette trade so far and how this could be improved.

This study comprised desk-based mapping of existing documents (‘grey literature’) (n=24) (due to the presence of only one academic literature published) and semi-structured stakeholder interviews (n=10) either via web-conferencing or face-to-face.

A mapping of unpublished ‘grey’ literature was conducted to identify materials with information on the scale and nature of the illicit cigarette trade in Ghana and the government’s response to this trade. We considered all documents dated 2012 to 2023 to give us a more comprehensive search to include documents from when Ghana passed the Tobacco Control Act. Searched materials included briefing documents, reports from government and non-governmental organizations (NGOs), blogs, commentaries, press releases, print press, and specific Twitter accounts to find studies on illicit cigarettes in Ghana. The mapping was conducted to better understand the nature of the illicit cigarette market in Ghana by identifying: 1) government policy in response to illicit cigarette trade, and 2) existing estimates of the scale of the illicit cigarette trade in Ghana (Supplementary file Materials 2 and 3).

Stakeholder interviews provided additional understanding of: 1) the extent and nature of the illicit cigarette market in Ghana, and 2) the tobacco industry’s potential involvement in/influence over this market. The interviews took place between January and June 2021. After consultation with tobacco control experts and discussions among the authors, we purposively recruited and interviewed ten individuals representing government agencies (Ministry of Health and Finance), Ghana Revenue Authority (GRA), Civil Society Organizations (CSOs), Media, and international organizations such as the World Health Organization (WHO). Interviews explored Ghana’s tobacco policy development, context and content of the policies, data sources for illicit cigarettes, evidence of industry interference, barriers, and facilitators to implementation of the illicit cigarette trade, and recommendations for tackling illicit cigarette trade in Ghana using an interview guide (Supplementary file Material 4). All interviews were transcribed verbatim to Word files and saved with identification codes on password-protected servers. Each interview lasted approximately 45 minutes and was later translated and transcribed. The final data interpretation was discussed among AS, FD, and AG and also sense-checked for validity and clarity with three key informants representing CSOs and the GRA.

Transcripts were uploaded into the qualitative data management software NVIVO 12 and coded using a coding frame developed by AS, AG, and FD. Relevant data from the documents were extracted using a data extraction template capturing data on the name and source of data, topic, overview of key findings, and limitations. We then used an inductive and deductive thematic content analysis to code the transcripts and documents line-by-line, guided by the key research objectives. We then identified several themes and summarized the findings from the document review and stakeholders’ perceptions of the illicit cigarette trade in Ghana. A total of 52 data sources were identified, of which 24 were eligible for extraction (mainly online print press and Twitter).

The search strategy and data sources are in the Supplementary file Materials 2 and 3.

## COMMENTARY

The findings from the mapping exercise and stakeholder interviews are presented under five key themes: 1) Government response to illicit cigarette trade; 2) awareness and extent of illicit cigarette trade in Ghana; 3) drivers of illicit cigarette trade; 4) tobacco industry (TI) influence/involvement on policy and illicit cigarettes; and 5) recommendations for addressing illicit cigarette trade in Ghana (Table 1). A summary of the findings for each theme is provided below.

**Table 1:** Summary of themes and subthemes derived from the qualitative interviews and mapping exercise

**Theme 1**	**Government response to illicit cigarette trade**
**Theme 2**	**Awareness and extent of illicit cigarette trade in Ghana**
**Theme 3**	**Drivers of illicit cigarette trade** Porous borders Lack of political interest Poor awareness and capacity of tobacco control officials Tobacco tax structure
**Theme 4**	**Tobacco industry influence on policy and illicit tobacco** Arguments alleging complicity and/or counterproductive policy influence Arguments opposing complicity and/or counterproductive policy influence
**Theme 5**	**Recommendations for addressing illicit cigarette trade in Ghana**

### Theme 1: Government response to illicit cigarette trade

The majority of the documents identified from the mapping were mainly related to the seizure of illicit cigarettes by customs officials in Ghana^18-21^. While some seizures were from the northern part of the country around the unapproved borders, others were in the Ashanti Region of the country (south). The estimated cost of the cigarettes seized ranged from US$ 1000–10000 . As highlighted in one document:

*‘The Customs Division of the Ghana Revenue Authority (GRA) seized and destroyed foreign cigarettes and dried leaves suspected to be Indian hemp, estimated to cost GH*¢*136880, at the Fuo Landfill Site in Tamale.’*

Seizures from the southern zone of the country note a smaller value of the seized products:

*‘The Customs Division of the Ghana Revenue Authority seized and destroyed 500 boxes of cigarettes smuggled into the country valued at GH*¢*10000 with no health warnings on them. They were seized in parts of the Brong Ahafo and the Ashanti regions and were destroyed at the Kaase dumping site in Kumasi.*’

The government’s response to illicit cigarette trade was noted to be particularly slow compared to the country’s early ratification of the FCTC in 2004 and the passage of a Tobacco Control Act in 2012. Documents from the WHO country office and CSO reports revealed several calls to ratify the Protocol in 2015. A delay in the ratification process of the Protocol was noted in these documents despite Ghana being a signatory to it as early as 2013^22-24^. One press release indicated:

*‘The Ghana Country Director of the World Health Organization (WHO), Dr Magda Robalo, urged the Ghanaian government to ratify the protocol to eliminate illicit trade in tobacco products.’*

CSOs such as VALD also urged for the ratification of the Protocol:

*‘The programs director of Vision for Alternative Development (VALD), a non-governmental organization, has called on Parliament to ratify the protocol on illicit trade in tobacco*.*’*

In 2018, the GRA, in response to illicit trade, introduced excise tax stamps^25^. These tax stamps were designed with digital and other security features affixed on excisable goods to show that taxes and duties have been paid or would be paid ^25^. Stakeholders in the interviews also alluded to the introduction of the tax stamps but insisted on the need for a more robust country-specific track and trace system. The tax stamps were initially linked to industry tactics given the partnership between the GRA and De La Rue (a UK-based manufacturer of security-printed products), which was later suspended^26^. GRA had signed a five-year contract with De La Rue to partner with a Ghanaian print company (Buck Press) to create a track-and-trace excise tax stamp system. Still, De La Rue outsourced the track-and-trace system to Atos, which had extensive links to the industry and its tracking and tracing technology, Codentify^27^. Court documents also outline that the tobacco industries have supported track and trace in over 35 countries, of which Ghana is one^28^.

Further, a Needs Assessment on Tobacco Control in Ghana was also noted in the review^29^. This was under the FCTC 2030 project, a global initiative funded by the Governments of Australia, Norway, and the United Kingdom to support countries in strengthening WHO FCTC implementation to achieve the Sustainable Development Goals. Through the FCTC 2030 project, the Secretariat of the WHO FCTC, the United Nations Development Plan, and WHO Ghana stand ready to support the Government of Ghana to reduce the tobacco-induced social, economic, and environmental burdens by implementing evidence-based tobacco control laws and policies^30^. A key recommendation from the assessment was to combat the illicit tobacco trade in Ghana^31^.

### Theme 2: Awareness and extent of the illicit cigarette trade

Despite stakeholders alluding to the presence of illicit cigarette trade in Ghana, the mapping exercise points to a lack of data in terms of the scale of this. Only two estimates were identified, one was from an academic source using a single-stick survey, and the other was from Euromonitor International. Stakeholders interviewed were, however, not aware of Euromonitor’s estimates as a source of information for illicit cigarettes in Ghana, and the only data mentioned were those from the revenue department, which are not publicly available. Whilst some stakeholders quoted estimates of illicit cigarettes to be around 10% of the market:

*‘The Customs data is very reliable but maybe inadequate because that is the only one they are capturing through the approved routes. So, once it is approved, then it is reliable, but illicit trade in approved routes may end up about 10% (of the actual amount of illicit product).’* (Stakeholder, Government)

Others mentioned it to be between 10–15%:

*‘We could still have a high percentage of illicit cigarettes, but the revenue authority is not tracking well. I would say 10-15% illicit market in Ghana.’* (Stakeholder, International organization)

The lack of country-specific data on the illicit cigarette market was also a point of concern to some stakeholders:

*‘Illicit trade in tobacco products has existed over the years, and now, there has not been any research to support this, so I am not able to tell whether it has shot upwards or minimized; but the point is that we are still facing the illicit trade of tobacco products in the country and nothing much has changed.’* (Stakeholder, Civil society)

On the other hand, one stakeholder claimed that data on illicit cigarettes was present, which has not been analyzed to estimate the extent:

*‘Illicit tobacco trade is a big problem, and we don’t have data. What we have is some data from local data from 2004 to 2012, which is unpublished. But from that period, we have not got anything else in that regard.*’ (Stakeholder, International organization)

While there was a view that it was a ‘big’ problem, there was also a perception that the illicit trade market had shrunk in recent years due to the tightening of border controls, as indicated by customs officials.

### Theme 3: Drivers of illicit cigarettes tobacco

The mapping exercise and the interviews identified four main subthemes of factors that facilitated illicit tobacco trade: 1) porous borders (unapproved routes), 2) lack of political interest, 3) poor awareness and capacity of border officials, and 4) the tobacco tax structure of the country^32-35^.

*Porous borders*

Stakeholders described ‘porous’ borders and unapproved shipping routes as being particularly problematic, as well as a lack of enforcement staff to monitor the borders and carry out inspections. One stakeholder noted:

*‘Smuggling will continue in our region. Why? Because the border control is poor, very poor if you cross around even Togo you can see how it is so easy to enter with products up and down, coupled with corruption.’* (Stakeholder, Civil society)

According to Euromonitor and the Organized Crime and Corruption Reporting Project, the sale of illicit cigarettes in Ghana has been linked to porous borders around the northern border of Ghana with Burkina Faso and its eastern border with Togo^36^. Similarly, Singh et al. pointed to the Ghana-Togo and the Ghana-Burkina Faso borders as having the highest burden of illicit cigarette sales.

*Lack of political interest*

Stakeholders expressed views on a lack of political interest in terms of allocating funding or prioritizing the issues of the illicit cigarette trade. This was evident in some of the interviews where specific reference was made to the delay in the ratification of the Protocol as a clear sign of lack of government interest:

*‘The point is that the delay in the completion of the ratification process of the illicit tobacco protocol was said to be due to COVID-19; I mean, for God’s sake, some of them were ratified in October 2019 thereabout, and this pandemic came somewhere later than that, so what has prevented them from completing the ratification process?’* (Stakeholder, Civil society)

Civil societies in several press releases indicated the lack of government interest in the illicit cigarette trade in Ghana due to the delays in the Protocol ratification.

*Poor awareness and capacity of officials at the borders*

The general view of stakeholders was the lack of awareness of both the extent of the illicit cigarette trade and its impact. The FDA and customs officials indicated this lack of awareness. They indicated the lack of appropriate training in the illicit cigarette trade of customs officers and tobacco vendors who either did not know they were selling illegal products or were aware and selling them in locations that were easy to hide to avoid seizure:

*‘Illicit cigarette is mostly sold around the coast, villages, and around the beaches, and the retailers normally hide the illegal ones and display the legal ones. Also, some smokers don’t know the difference between legal and illegal cigarettes, and some retailers also hide them in handbags and sell them to their clients around the slums.’* (Stakeholder, Government)

Related to this view of a general lack of awareness was a stakeholder perception that there is very little media coverage of the illicit cigarette market and that the only aspects covered by media were seizure activities, many of which are under-reported. Our mapping exercise identified documents mainly on seizure activities by customs officials but scanty documents on other areas of cigarette-related customs activities^18-21,37^:

*‘The only area the media can provide information is when there is a seizure. When there is a seizure, you will hear of it, but for them to report on pure data I cannot remember the last time they did because most of the investigative journalism is on corruption and the cocoa smuggling. We have never benefited from a tobacco smuggling investigation.’* (Stakeholder, Civil society)

*Cigarette tobacco tax levels*

Stakeholders wrongly perceived current taxes in Ghana as one of the highest in the African region and a reason for illicit trade (Table 2). Despite evidence to the contrary, they also believed that these higher taxes could increase the demand for illicit tobacco:

**Table 2:** Excise taxes on tobacco products in Ghana for 2015 and 2023

*Product category*	*Tax rate as a percentage of the price CIF value (2015)*	*Excise duty Amendment Act (2023)*
Cigarettes	175	50% of the ex-factory price
Cigars	175	50% of the ex-factory price
Snuff and other tobacco	170.65	GHS 280 per kg
Electronic cigarettes	Not included	50% of the ex-factory price
Electronic smoking devices	Not included	50% of the ex-factory price

*‘The tax rate here is very high. The tax rate on excise duty alone, the rate is 175%; the highest in the region. So if somebody succeeds in cutting away with 175% of the value, which is even more than many other places, the value is a huge incentive for the people not to pay together with the other taxes, therefore would like to evade.’* (Stakeholder, Government)

### Theme 4: Industry interference in policy making and in controlling illicit cigarette tobacco trade

Despite substantial evidence of historical tobacco industry complicity in illicit trade^38^, there were mixed perceptions of the industry’s current role in influencing policy and illicit tobacco trade in Ghana among stakeholders. One stakeholder indicated the link was clear between illicit trade and industry:

*‘There is a direct link between the tobacco industry and illicit trade. Anytime you see illicit trade, whether, at the borders or the packaging, the industry is behind it because, in the end, somebody must make the big money, and the big money maker is the industry, and they will exploit every opportunity or every loophole to be sure that they get what they want.’* (Stakeholder, Government)

Stakeholders pointed to the historical involvement of the industry during the passage of the Tobacco Control Act and the Regulations and more recently during the passage of pictorial warning labels as an indication of the industry’s involvement in illicit trade:

*‘In Ghana, there is some industry interference more or less indirectly, but I have heard that the tobacco industry uses some organizations to infiltrate and interfere; they work behind the scenes - this happened when we wanted parliament to pass the legislative instrument. They were meeting with the Parliamentary Committee for Health at night. This affected some of the provisions in our Legislative Instrument.’* (Stakeholder, International organization)

Two stakeholders, however, noted the lack of any evidence of industry involvement and the rather supportive positioning of the industry toward measures to address the illicit cigarette tobacco trade:

*‘The tobacco industry, instead has paid so much and have complied to ensure that their products are in conformance with what should be marketed in the country. They don’t want to see competitive products that are not complying because those products are even cheaper. The tobacco industry even does their monitoring to check illicit products.’* (Stakeholder, Government)

### Theme 5: Recommendations for combatting illicit cigarette tobacco

This section highlights the recommendations made by the stakeholders and findings from the document mapping. Due to the lack of country-specific data, stakeholders emphasized the need for robust studies. Also, they expressed an urgent need to implement the Protocol and develop an efficient track and trace system along with a task force that includes representation from the police, customs immigration, FDA, and media. One participant summarizes the recommendations:

*‘We need to get the protocol ratified, that is the first thing, we also need to develop our own tracking and tracing system, and we need to orient our law enforcement agencies such as the customs, police, and immigration. We also need a data collection tool that we can track properly. Currently, we are not tracking effectively, we also need solid evidence, when we seek the Government’s input to get their support for which we need data.’* (Stakeholder, International organization)

In one stakeholder meeting, the GRA documented key recommendations that aligned with the interviews conducted^39^. These include the need for regular border patrols, joint cross-border consultative meetings, exchanging information through the Economic Community of West African States (ECOWAS), Interconnected Transit Freight Management System, forging close collaborations with competent state agencies, and rejection of all forms of interference with the tobacco industry in government policy discussions.

Stakeholders (mainly from customs and the revenue authority) noted that for the effective implementation of the Protocol, porous borders and individual duty-free allowances (imported tobacco products up to 1 pound [0.45 kg], should be addressed). The needs assessment document from the FCTC 2030 project also raised concerns about the seemingly minimal transparency in the system in disposing of all confiscated illicit tobacco products. Recommendations emerging from the document pointed to the need for increasing resources and technical capacity for border officials in enforcing appropriate penalties when tobacco is illegally brought into Ghana and rewards for ‘whistle-blowers’ who provide insight on illegal tobacco products^31^. A summary of the recommendations emerging from the interviews and the review follows:

- Need for more country-specific data on the extent of illicit cigarette sales.
- Develop an efficient track and trace system independent of industry.
- A working group task force for full protocol implementation.
- Regular border patrols and joint cross-border consultative meetings.
- Increasing resources for border officials and rewards for ‘whistle-blowers’.
- Capacity building, and information awareness campaign for custom, enforcement officials and government officials.
- Transparent confiscation and destruction of seized illicit tobacco involving media.
- Accelerated implementation of Article 5.3 of the FCTC.

This is the second study carried out in Ghana to provide insight into the extent and nature of the illicit cigarette trade. The study’s findings suggest that, while the Ghanaian illicit cigarette market is under-studied, its size likely ranges between 10% to 35%. There were several reports concerned with seizure activities conducted by the authorities^19-21,40^; these data did provide complete estimates of Ghana’s illicit tobacco trade. Stakeholders interviewed also noted that the illicit market in Ghana is currently about 10%, which they claimed to be high; however, 10% is a normal tax evasion that could be managed at the country level, which the authorities should not worry about^41^. The main contributing factors for the illicit cigarette market in Ghana were identified to be porous borders, a lack of political interest, industry influence, insufficient capacity, and awareness levels among customs officials, as well as the current tobacco tax structure. Nonetheless, the introduction of tax stamps (2018), the ratification of the Protocol (2021), and the drafting of an Excise Tax Amendment Bill (2023) were notable steps taken by the government to address the issue in Ghana.

The trade of illicit cigarettes has long been associated with Ghana’s porous borders, particularly its northern border with Burkina Faso and its eastern border with Togo. Singh et al.^[Bibr cit0011]11^ in their study found that sales of illicit cigarettes were higher at the border towns in Ghana, corroborating with the stakeholder views in our study. Euromonitor reports also pointed to the fact that Ghana’s major source countries for illicit cigarettes were Burkina Faso and Nigeria, with Nigerian products smuggled in via Togo. Likewise, over 40% of illicit cigarettes identified in the study by Singh et al.^[Bibr cit0011]11^ were from Togo. About half of the parties to the Protocol (belonging to mainly LMICs) lack the technical capacity of customs and enforcement authorities to successfully implement the Protocol at the global level^42^. These findings reinforce the need for strengthening patrolling and border control in addition to building capacity and training for authorities belonging to customs and police (as highlighted by some stakeholders).

Given that the link between cigarette tobacco taxation and smuggling is doubtful and inconsistent, stakeholders still attributed high excise taxes on cigarette products in Ghana (compared to other neighboring West African countries) as a major driver of the illicit cigarette tobacco trade in Ghana. Taxes in the African region in general, and Ghana in particular, are relatively low compared to all other parts of the world^43^. Contrary to stakeholders’ views, independent research has been much more doubtful of suggestions that tobacco taxation drives smuggling. In Ghana, the total excise tax on tobacco products accounted for only 31.8% of the average retail price (before the recent passage of the Excise Tax Amendment Bill 2023). Although cigarette prices, particularly in instances where there are price discrepancies between nearby locations, may play some role in motivating some cross-border illicit trade, the scale of such trade is usually insignificant and does not support the industry narratives that tax increases will, by default, lead to an increase in illicit trade.

Further, contrary to tobacco industry arguments, taxes and prices have only a limited impact on the illicit cigarette market share at the country level, and Africa provides an appropriate example as it has both a low level of tobacco taxation and low tobacco prices compared to Europe, and higher levels of tobacco smuggling. Tobacco taxes have generally been lower in the ECOWAS (including the West African Economic and Monetary Union) countries than the global average and the average for SSA. Indeed, the current strengthening of the tobacco tax design for tobacco in Ghana (Excise Duty Amendment Bill 2023) is a positive move towards reducing smoking and generating revenues to address economic and public health burdens.

An example of successful tobacco taxation for public health in the African region can be observed in South Africa. In ten years of tobacco tax increases beginning in 1994, consumption fell by 36%, saving thousands of lives and increasing tax revenues for social programs^44^. Our stakeholder interviews also indicate the pressing need to improve stakeholders’ awareness and knowledge levels via information campaigns and workshops on the tax structure for Ghana and SSA.

Further, the role of the tobacco industry in the illicit trade of tobacco products has been documented earlier^45^. In Africa, several historical documents demonstrate smuggling as an important component of BAT’s market entry strategy to gain leverage in negotiating with governments for tax concessions and gain a market presence. Stakeholders had mixed views on industry involvement and were not fully aware of the tactics of the industry, despite clear evidence of their role in perpetuating illicit tobacco in several countries. Previous research analyzing internal tobacco industry documents from BAT has revealed industry involvement in smuggling in at least 40 of the 54 African countries. BAT has also been known for using bribery and corruption to influence politicians, parliamentarians, public health officials, and even staff of competitor tobacco corporations in several countries in the region, such as Uganda, Rwanda, Burundi, Kenya, Comoros, and Ghana. A clear observation in Ghana was the interference by industry (particularly from alcohol industries) during the recent passage of the Excise Tax Amendment Bill (2023). Also, the 2021 and 2023 Tobacco Interference Index Report ranked countries in SSA according to how much influence the industry wielded over them. Zambia topped the list for the highest level of interference, followed by Tanzania, South Africa, and Mozambique^46,47^. Ghana was found to have 56 and 58 points in tobacco industry interference in 2021 and 2023, respectively^46,47^. Tobacco companies also continue to provide funding for public-policy think tanks, such as the IMANI Center for Policy & Education in Ghana, which has publicly opposed tobacco control and even the link between smoking and lung cancer^48^. Stakeholders thus indicated the need for a track-and-trace system independent of industry influence as outlined in the Protocol. Likewise, in the recent progress reporting cycle of the Protocol^42^. Also, there is a need for advocacy efforts focusing on enforcement and implementation strategies targeting the industry tactics for full implementation of the Protocol.

### Limitations

This study has some limitations. First, we could not reach all the policymakers who participated in formulating the tobacco policies due to the COVID-19 pandemic. Nevertheless, we had a good mix of representation from the government, CSOs, media, and international organizations. Secondly, the results obtained in this study may not be generalizable to other countries due to Ghana’s unique cultural and political scenario that shapes the policies’ context and content. Finally, there is no guarantee that the opinions expressed by the interviewees are fully consistent with the views of all the representatives of given communities or organizations. To address this limitation, we incorporated some sense-checking of our emerging analysis with three key stakeholders (see the Methods section).

## CONCLUSION

The issue of illegal cigarette trade in Ghana appears to be a cross-border issue. Following Ghana’s ratification of the Protocol, the next steps will be implementing the Protocol’s Articles. However, our findings indicate a lack of financial resources and knowledge needed to apply the Protocol. Stakeholders prioritized the need for more credible scientific data on illicit tobacco and more investment in border patrol capabilities, including training and capacity building for customs and government officers on tobacco pricing and industry influence on legislation.

## Supplementary Material



## Data Availability

The data supporting this research are available from the authors on reasonable request.
