# Supplementary material for: The trade of illicit cigarettes in Ghana: Insights from a policy synthesis and qualitative study
Source: Tob Prev Cessat. 2025 Jan 20;11:10.18332/tpc/195578. doi: 10.18332/tpc/195578 (PMC11744211; doi:10.18332/tpc/195578)
Supplement: Supplementary file 1 [file TPC-11-08-s1.pdf]

Supplementary file 1: **Tobacco use in Ghana**

Prevalence (15+ years): Males (5.5%), females (0.3%) (2019)
Youth smoking (10-14 years) males (4.4%), females (5.5%) (2019)
Tobacco related mortality: 6639/year
Local tobacco product manufacturing: No
Tobacco farming: No (small-scale, not commercial)
Tobacco products: mainly cigarettes from imported from British American Tobacco (Nigeria)
Tobacco tax structure: Mixed (specific/Ad Valorem)
Economic cost of smoking and tobacco use in Ghana: 1,215,801,856 Ghana cedis/year
Focal point for tobacco control in Ghana: Food and Drugs Authority
National Tobacco Control law: Pubic Health Act 851

[Source: WHO report on the global tobacco epidemic. 2023 & Singh A, Owusu-Dabo E, Dobbie F, et al. A situational analysis of tobacco control in Ghana: progress, opportunities and challenges. J Glob Health Rep. 2020;4. doi: 10.29392/001C.12260]

Supplementary file 2: Summary of approach and data sources

Three rounds of data searches were conducted. The collected material was limited to the period from 2012 (after the passage of the Tobacco Control Act in Ghana) to December 2023. The first search was conducted on 15 July 2021, the second on 31 July 2023, and the final on 15 January 2024. **Search terms**

Searches were conducted using the Google search engine and Nexis (a resource for searching digital copies of broadsheet and tabloid newspaper articles). The terms 'illicit', OR 'counterfeit', OR 'contraband', OR 'illegal', OR 'industry', OR 'companies', 'smuggling', in combination with cigarettes AND Ghana were searched in the "All" section of Google and the "News" section of Google. Any articles that appeared to address illicit trade and cigarettes for the Ghana were assessed against the inclusion criteria for data extraction.

Inclusion criteria

- Is it relevant to at least one aspect of the mapping research objectives? (See section on desk-based mapping)
- is this data or report funded by the tobacco industry? (excluded if it was) (via investigative searching or by looking for a funding disclosure in the selected reports).
- Does this data fit the search date from 2013 till 2023

• **Website search:** Ghanaian Ministry of Information website, the Ghanaian Ministry of Health website, the WHO Country Office for Ghana website, WHO FCTC and NGO websites: Centre for Tobacco Control Africa, AMREF Health Africa, African Tobacco Control Alliance, Tobacco Atlas and Ghana Non-Communicable Disease Alliance.

• **Online print press** using Google alerts and Nexis (alerts include☺

•**Twitter accounts** for Ghanaian Ministry of Health, Ghanaian Ministry of Information, WHO Ghana, WHO FCTC, and the Ghana NCD Alliance

• **Consultation** with colleagues in Ghana

Number of data sources identified: 52

Number eligible for data extraction: 24 (mainly online print press and twitter)

© 2025 Singh A. et al.
